# A New Water Level Measurement Method Combining Infrared Sensors and Floats for Applications on Laboratory Scale Channel under Unsteady Flow Regime

**DOI:** 10.3390/s19071511

**Published:** 2019-03-28

**Authors:** Samer Majdalani, Jean-Philippe Chazarin, Roger Moussa

**Affiliations:** 1 Laboratoire HydroSciences Montpellier, Centre National de la Recherche Scientifique, Institut de Recherche pour le Développement, Université de Montpellier, 34090 Montpellier, France; jean-philippe.chazarin@umontpellier.fr; 2 Laboratoire d’étude des Interactions entre Sol-Agrosystème-Hydrosystème, Université de Montpellier, Institut National de la Recherche Agronomique, Institut de Recherche pour le Développement, SupAgro, 34090 Montpellier, France; roger.moussa@inra.fr

**Keywords:** water level measurement, laboratory scale channel, infrared remote sensor, float, unsteady flow regime, loop rating curve

## Abstract

In this paper, we studied water transport under an unsteady flow regime in an experimental channel (4 m in length; 3 cm in width). Our experiments implicated some measuring requirements, specifically, a water level (WL) detection technique that is able to measure WL in a range of 2 cm with a precision of 1 mm. The existing WL detection techniques could not meet our measurement requirements. Therefore, we propose a new measurement method that combines two approaches: An “old” water contact technique (float) with a “new” remote non-contact technique (infrared sensor). We used an extruded polystyrene (XPS Foam) that needed some adequate treatment before using it as float in experimental measurements. The combination of IR-sensors with treated float foam lead to a sensitive measurement method that is able to detect flat and sharp flow signals, as well as highly dynamic variations of water surface level. Based on the experimental measurements of WL and outflow at the channel output, we deduced a loop rating curve that is suitable with a power law adjustment. The new measurement method could be extended to larger scale applications like rivers and more complicated cross section geometry of irregular shape.

## 1. Introduction

In hydraulics and hydrology, it is important to monitor water level (WL) in rivers and channels in order to understand flood propagation and prevent the consequent damages on neighboring human installations and activities. In hydrological studies, researchers generally have a main difficulty in validating their models which is the lack of in-situ data. In-situ data in hydrology is difficult to attain for various reasons (scale of observation, difficult access to the zone of interest, dangerous access during extreme events, unpredictable timing of the event, human and material cost, etc.). Therefore, hydrology/hydraulic researchers are increasingly relying on laboratory scale experiments, where the different models hypothesis can be tested under controlled conditions. In addition, laboratory scale experiments permit to do several replicates in short time periods, which is necessary to obtain reliable and reproducible experimental results. Some examples of hydrological studies on laboratory scale experiment are the impact of land use change and river management on floods [1], the impact of dikes on inundation [2], and the impact of vegetation on hydraulic resistance [3,4]. 

To understand water transport during unsteady flow events, we conceived an experimental channel (4 m length, 3 cm width, 5 cm height) where water flow is well controlled at the input and well measured at the output. The unsteady flow scenarios that are tested on the experimental channel will serve as database for candidate hydrological or hydraulic models. The dimensions of the experimental channel were chosen so that the following constraints are respected:

(C1) The consumption of deionized water and solute is reduced to its minimum, which explains the relative small values of width and height.

(C2) The experimental duration is as short as possible, so that many replicates of a same flow scenario can be done, which leads to accurate and reliable experimental curves.

(C3) The space occupation of the experiment is reduced, which was achieved by constructing the channel in a serpentine way (four juxtaposed 1 m conduits). This also reduces the experimental cost of water tubes and electrical cables. 

According to the latter constraints, water height in the experimental channel can vary between 0.5 cm (threshold height) and 2.5 cm (the most extreme flow scenario), which gives a range of WL variation of 2 cm. One of the questions that we had to answer in our work is the following: “What kind of technique would permit us to measure WL in a range of 2 cm with a precision of (at least) 1 mm?”

We started by exploring the existing WL measurement techniques. These techniques can be subdivided into two main categories: water contact and remote sensing (non-contact). Traditionally, water contact approaches were the most used techniques, and they were implemented on hydrological stations. Though, with the development of modern technologies, remote sensing techniques are becoming a widespread alternative. Under the water contact category, we find:

- Pressure measurement. It is an old technique, yet it is still in use. It is based on the measurement of hydrostatic pressure to deduce water height above the pressure detector/sensor.
- Float gauge. It is an old technique that is based on the movement of a floating object which is related to a mechanical system that deduces the position/height of the float, and thus the water surface level [5].
- Fiber optic. Fiber optical sensors have recently been used to detect water depth and temperature [6,7,8,9]. They are used in many domains such as the oil and gas industry, marine applications, and biomedical applications.

Under remote sensing (non-contact) category, we find:

- Radar (Radio Detection and Ranging). Radar is a remote sensing instrument that uses radio waves to detect objects. Initially used to detect airplanes during WWII, it is now also used in meteorology and hydrology to measure water level variations on large-scale areas like lakes, forests, flood plains, and large rivers basins [10].
- LIDAR (Light Detection and Ranging). LIDAR is a remote sensing instrument based on laser emitting pulses. It was first developed for meteorology applications (measure of cloud heights) and atmospheric observations [11]. It is now used in other domains to track moving objects like pedestrian tracking [12] and cargo handling in ports [13], as well as measuring water depth over lakes via unmanned aerial vehicle (UAV) [14].
- Ultrasonic waves. Ultrasonic sensors are very commonly used to detect variations in water surface level. They can be fixed on a flying object like UAV or an existing infrastructure like a bridge over a river [15,16,17].
- Infrared sensors. Infrared sensors are not commonly used for surface water detection because the beam emitted by the sensors needs to be interrupted by a non-transparent edge, which is not the case with water. However, this technique was found to be useful in detecting debris flows since the flowing mass can interrupt the beam [18].
- Image/video. Remote video surveillance images are recently used to detect water limits/edges in order to deduce water level variations [19]. Imaging is also coupled with the structure from motion technique in environmental mapping applications [20,21,22].

To answer our question regarding the suitable technique for measuring WL (in a range of 2 cm with 1 mm precision) at different positions/abscissa in our 4 m experimental channel, we noticed that:

Radar, LIDAR. Those techniques are suitable for large-scale measurements (in nature) rather than laboratory-scale experiments.

Imagery. Imagery could have been an alternative technique by detecting surface water edge from side photos. However, the serpentine form of our experimental channel makes it that only the outer conduits are accessible on the side to camera. The inner conduits are bounded on the side by the outer conduits, and they are only accessible to camera from the top.

Ultrasonic waves. The size of our experimental channel is not suitable for ultrasonic techniques that would work better on larger scales. We made some preliminary tests, and we found that the sensitivity of ultrasonic sensors could not meet our measuring requirements.

Pressure measurement. We recall that the flowing section in our experimental channel varies between 0.5 cm × 3 cm (1.5 cm^2^) and 2.5 cm × 3 cm (7.5 cm^2^). It is thus a relatively small section, and it is preferable not to introduce big sensors inside it for measuring purposes (like pressure sensors) because the sensors would be big enough to disturb flowing water and consequently the shape of the surface water level. Moreover, pressure sensors are relatively expensive according to our restricted budget.

Fiber optic. Our experimental channel is dedicated to study solute transport during unsteady flow events, and it is well known that the change in water density would affect the refraction index and thus the WL measurement by fiber optic technique. The refraction index also varies with temperature, and our experimental study is not dedicated to operate under constant temperature conditions. In addition, we were not sure if the sensitivity/precision of the fiber optic technique would meet our requirements. [6] reported a 2.5 cm accuracy, although new techniques are being developed to improve such accuracy [7,9].

Therefore, we are left with the infrared technique. Infrared sensors have the advantage of being relatively cheap, and they are at the same time highly sensitive. We chose infrared sensors with integrated signal amplifier and signal treatment device, so that there is no need for supplementary electronic devices cost. They are small enough to be implemented on several positions/abscissa along the 4 m experimental channel, and they do not interfere with each other. Thus, they are suitable for our laboratory-scale experiment. As other remote sensing techniques, the WL measurement/detection is based on the reflection of the incoming beam (from the emitter cell) at the water surface and the detection of the reflected beam (by the detector cell) at a later time interval. Our first attempts with infrared sensors were not successful because the beam reflection at the water surface did not occur—the beam was instead reflected by the channel floor. Then, we tried to change the color of the channel floor, but the result remained the same. After that, we tried to color the water in the channel (by a dye) hoping that this would improve the reflection at the water surface, but the result was still the same—beam reflection at the channel floor, instead of the water surface. 

To overcome this problem, we decided to “dress” the water surface by putting an obstacle at the water surface that would prevent the infrared beam from going into the water (towards the channel floor) and would force the entire beam reflection at the water surface. At the same time, such an obstacle must not disturb the flowing water (as we argued when we rejected the pressure sensors option). So, the obstacle must be:

- Hydrophobic. It should not interact with water chemically or physically, so that no contamination of flowing water occurs when we study solute transport during unsteady flow events.
- A lightweight float. It should stay at the water surface without any submersion, and it should instantly follow the moves/variations of water surface. Therefore, its level reflects exactly the surface water level.
- A reflector. It should entirely reflect the incoming infrared beam.

We found that the material that meets the aforementioned requirements is the extruded polystyrene (XPS Foam). Due to XPS foam properties and to infrared high sensitivity sensors, we were able to address our measuring requirements: a WL range of 2 cm with 1 mm precision over 8 different positions/abscissa along the 4 m experimental channel. 

The new WL measurement method proposed in this paper combines in fact one “old” water contact technique (float) with a “new” remote non-contact technique (infrared sensor). For a laboratory scale experiment, our new measurement method is more suitable than other existing techniques. The new WL measurement method was tested on several unsteady flow scenarios, and it gave satisfactory results as it was able to detect flat and sharp flow signals, as well as highly dynamic variations of water surface level. The paper is organized as following: Section 1—Introduction, Section 2—Material and method, Section 3—Results and discussion, and Section 4—Conclusion.

## 2. Material and Method

### 2.1. Infrared Sensor

The IR-sensor used in our experiments is GP2Y0A41SK0F model (SHARP Corporation). It is a distance measuring sensor unit with a distance range between 4 cm and 30 cm. The IR-sensor has a mass of 3.6 g, a size of 29.5 mm × 13.0 mm × 13.5 mm, and it delivers an analog output type signal. A combination of PSD (position sensitive detector), IR-LED (infrared emitting diode) and signal processing circuit are integrated within the sensor. The adoption of a triangulation method makes the distance detection almost independent of the object reflectivity, the environmental temperature and the operating duration.

**Figure 1:**
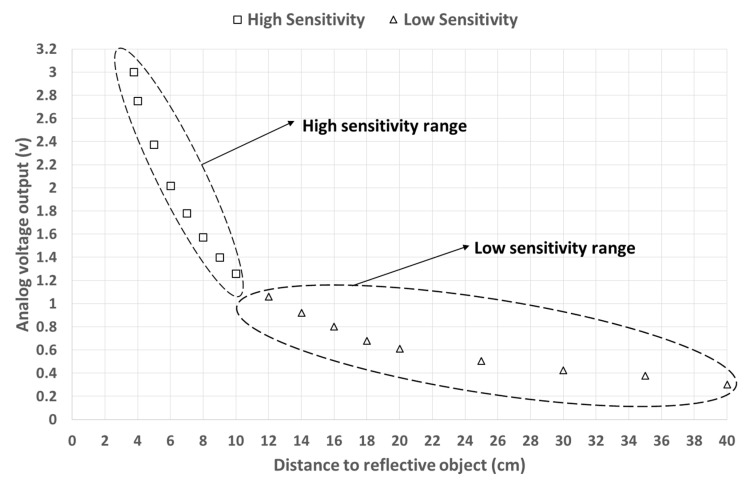
Infrared sensor response as a function of the distance to reflective object. The curve is based on the constructor (SHARP Corporation) specification manual for an object covered with white or gray paper. Squares: high sensitivity range. Triangles: low sensitivity range.

The IR-sensor sensitivity curve is given in Figure 1 according to SHARP specification manual. It represents the variation of the sensor output signal as a function of the detection distance variation (between 4 cm and 40 cm). The curve is almost the same for both white paper (reflectance ratio 90%) and gray paper (reflectance ratio 18%). We can distinguish two zones on the sensitivity curve—a steep variation zone and a flat one (Figure 1). 

**Figure 2:**
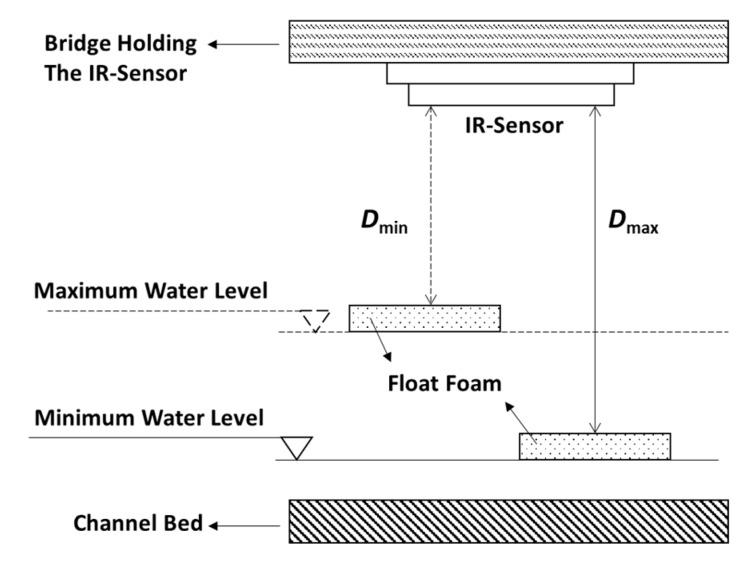
The distance between the IR-sensor and the float foam varies between *D*_min_ (respectively *D*_max_) for a maximum (respectively minimum) surface water level. In our flow simulation experiments, we would have *D*_min_ = 6 cm and *D*_max_ = 8 cm.

The steep (respectively flat) variation zone corresponds to the domain (or distance range) where the IR-sensor sensitivity is high (respectively low). The high sensitivity range is roughly between 4 cm and 10 cm. In our flow simulation experiments, the minimum and maximum flows could lead to surface WL variations between 0 cm and 2 cm. Therefore, we installed the IR-sensor on a bridge, so that the distance between the float foam and the sensor varies between 6 cm and 8 cm (Figure 2). The measurement precision of the IR-sensor is 1 mm, which respects our measuring requirements.

### 2.2. Float Foam

**Figure 3:**
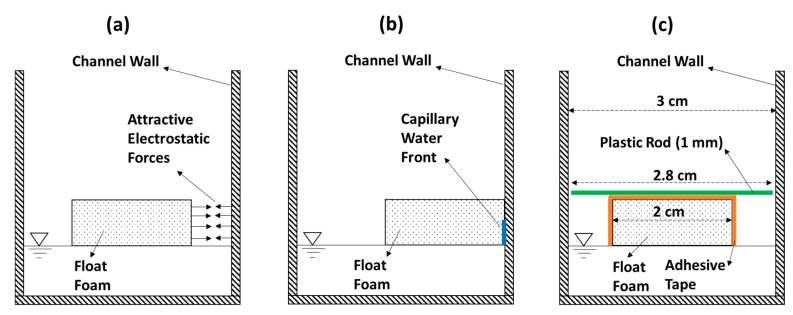
(**a**) Attractive electrostatic forces between the float foam and the channel walls. (**b**) The float foam is attracted to the channel wall and a capillary water front (blue color) is developed in a thin film at the float-wall interface. The resulting capillary forces stick the float to the channel wall. (**c**) Treatment of float foam. An adhesive tape (orange color) is applied on all float surfaces that are in contact with air, which reduces the attractive electrostatic forces. A thin plastic rod (green color) is installed at the back and front edges of the float foam. It keeps the float foam away from the channel walls and thus impedes the development of attractive capillary forces. The thin plastic rod is 1 mm in diameter and 2.8 cm in length.

The float foam is an extruded polystyrene (XPS Foam). It has a parallelepiped form: 10 cm in length, 2 cm in width, and 1 cm in height. The float is lightweight (density around 35 kg/m^3^) and hydrophobic, but at the same time, it is attracted by electrostatic forces to the experimental channel walls (made of Polymethyl Methacrylate—PMMA extruded) as shown in Figure 3a. When the float foam touches the channel wall, a thin capillary water front develops at the float-wall interface (Figure 3b). 

Due to the resulting capillary forces, the float will stick to the channel wall, and will no more follow the variations of the WL in the channel. The lightweight property of float foam makes it so that the float weight could be less than the capillary forces that stick it to the channel wall.

**Figure 4:**
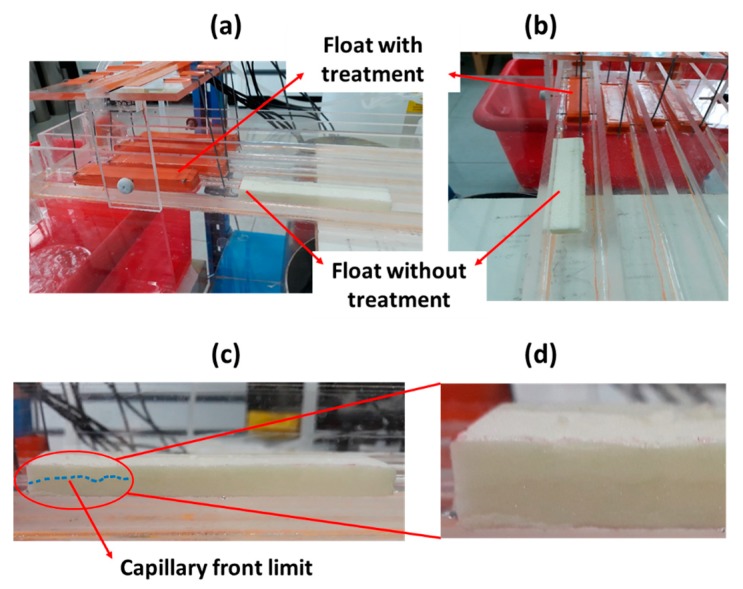
(**a**) Comparison between two float foams with or without treatment. (**b**) The attractive electrostatic forces drive the float foam to the channel wall. (**c**) A sketch of the capillary water front (dashed blue) that develops at the foam-wall interface when the float foam sticks to the channel wall. (**d**) Zoom of the capillary water front.

In order to overcome this problem, a special treatment should be done to the float foam. We applied an adhesive tape on all float surfaces that are in contact with air so that the attractive electrostatic forces (between float foam and channel wall) are highly reduced. Yet, there is still another problem. If the surface water perturbations drive the float to the wall, capillary forces would always develop. In order to avoid such situation, the float must be kept away from the channel walls. We succeeded to do that by installing at the back and front edges of the float a lightweight thin plastic rod (1 mm in diameter and 2.8 cm in length) as shown in Figure 3c. A comparison between two float foams with or without a treatment is given in Figure 4, where we can see how the treatment keeps the float at the center of the channel (directly under the IR-sensor) and away from the walls.

### 2.3. Experimental Channel

**Figure 5:**
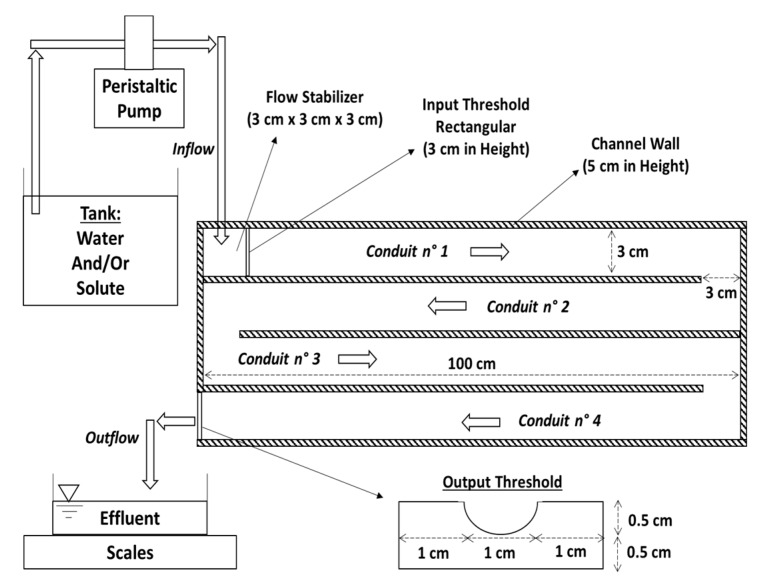
The experimental channel has a serpentine shape: it consists of four juxtaposed 100 cm conduits. The arrows show the flow direction: from water/solute tank, to peristaltic pump, to channel (input/inflow and output/outflow), and to effluent recipient.

In order to optimize the use of space, the experimental channel was conceived in a serpentine shape with four juxtaposed conduits of 100 cm in length and 3 cm in width (Figure 5). The conduits are separated by walls of 0.5 cm in thickness and 5 cm in height. A peristaltic pump (Gilson Minipuls 3, Gilson International, Villiers-le-Bel, France) controls water/solute flow according to a well prescribed unsteady flow scenario. We developed a special “pump control device” (based on Arduino Uno) that is able to vary automatically each second the rotation speed of the pump (and thus its flow). The prescribed unsteady flow scenario must be first converted into voltage information and saved on a SD card. Then, the SD card is introduced in the “pump control device” where the voltage information is sent to the pump via its external command terminal. 

At the channel input there is a small stabilizing chamber that reduces the development of waves related to the pulses of peristaltic pump. The stabilizing chamber has a 3 cm rectangular threshold. At the channel output, a 0.5 cm threshold (circular and rectangular) leads the water to an effluent recipient laying on scales. The mass measurement of the effluent is done each second, and it permits to deduce the outflow curve *Q_Out_* (*t*) at the channel output (abscissa 4 m) that is the response to the inflow curve *Q_In_* (*t*) imposed by the peristaltic pump at the channel input (abscissa 0 m). The scales mass measurement are saved on a Campbell Scientific CR1000 Data Logger.

**Figure 6:**
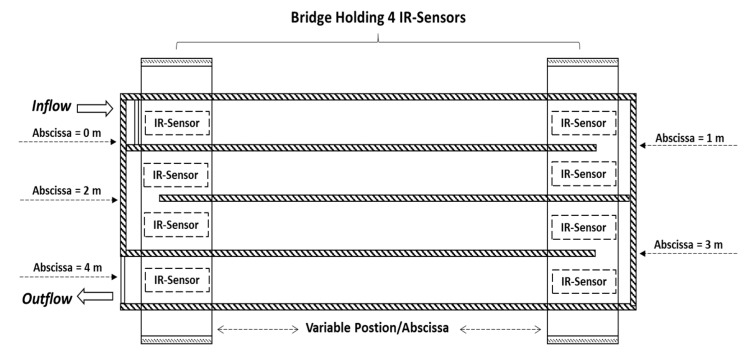
The experimental channel is equipped with two bridges each holding four IR-sensors. The bridges can be easily displaced to any desirable position/abscissa.

In order to measure the WL at different positions/abscissa of the experimental channel, we constructed two small bridges that can each hold four IR-sensors (Figure 6). 

The bridges can be easily displaced or translated to any desirable abscissa (from 0 m to 4 m) across the channel. Due to the serpentine shape of the experimental channel, the four IR-sensors that are mounted on a bridge can deliver WL measurements for wide abscissa ranges—a minimum of 2 m abscissa range when the bridge is at the right hand side in Figure 6, and a maximum of 4 m abscissa range when the bridge is at the left hand side in Figure 6.

In order to save the WL measurements (*H* (*t*) curve), we developed a special “IR data device” that is commanded by a software, and that stocks each second on a SD card (based on Arduino Mega) all the measurements coming from all the eight IR-sensors. All electronic devices are synchronized—same time reference for Campbell Data Logger, “pump control device”, and “IR data device”. The time interval considered in all these devices is one second.

### 2.4. IR-Sensors Bridges

**Figure 7:**
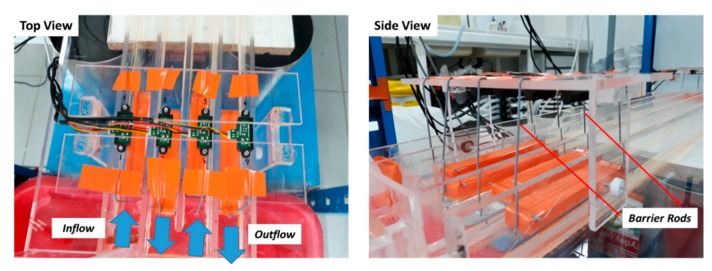
Top and side views of a bridge holding four IR-sensors. The blue arrows show the direction of water flow in the experimental channel. On both sides of the bridge, barrier rods are installed to keep the float foams at a standing still position below the IR-Sensors.

The float foam treatment guaranties that the float is kept in the center of the experimental channel (away from the channel walls). However, there remains a problem, which is that the float can navigate and that it can easily be driven by the current of the flowing water. In order to obtain a precise WL measurement by IR-sensor, the float must be kept at a standing still position below the IR-sensor. This was done by installing barrier rods on both sides of the IR-sensor bridges as shown in Figure 7. 

A major difficulty in the float treatment is to eliminate friction between the float and the barrier rods. That is why we chose rods that are made of plastic with very smooth surface. More, the adhesive tape that covers the float also has a very smooth surface. So theoretically, friction is reduced to its minimum. However, to be sure that friction does not intervene during the vertical displacement of the float, we made several manual measurements with a graduated ruler during the extremist tested unsteady flow event (Sc2a in Section 2.5) and we found that the manual measurements are the same as those given by the IR-Sensor, whether for low, intermediate or high flow intensities.

**Figure 8:**
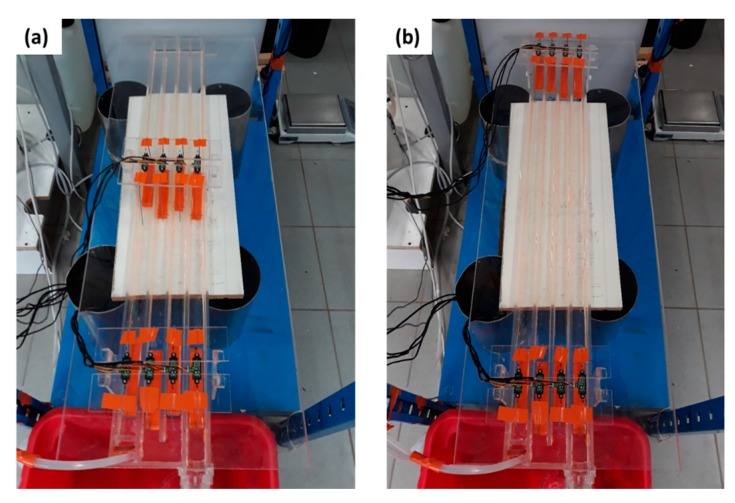
Two bridges holding each four IR-Sensors that can measure WL in the experimental channel at different abscissa. (**a**) For abscissa: 0 m, 0.5 m, 1.5 m, 2 m, 2.5 m, 3.5 m, and 4 m. (**b**) For abscissa: 0 m, 1 m, 2 m, 3 m, and 4 m.

Figure 8 shows a global view of the experimental channel equipped with two bridges, each holding four IR-sensors. In our experiments, we opted for the configuration of Figure 8b where the IR-sensors measure the WL at the abscissa 0 m, 1 m, 2 m, 3 m, and 4 m. 

### 2.5. Unsteady Flow Scenarios

We tested two unsteady flow scenarios in order to evaluate the efficiency of the WL measurement method proposed in this paper. The first scenario (Sc1) is based on the Hayami flood function [23]. The second scenario is based on a sine-squared function with three successive wave crests. Here are some mathematical specifications of both scenarios:

*First scenario* (Sc1)

The Hayami flood function is given by:

(1) $$Q_{In}\left(t\right)=Q_{p}+Q_{b}\frac{e^{z\left(2-\left(\frac{t}{\theta }\right)-\left(\frac{\theta }{t}\right)\right)}}{{\left(\frac{t}{\theta }\right)}^{1.5}}$$

where

*t* (s): time
*z*: shape parameter (taken 3)
*θ* (s): shape parameter
*Q_b_* (mL/s): base flow (taken 11 mL/s)
*Q_p_* (mL/s): permanent flow regime (taken 3 mL/s)
*Q_In_* (*t*) (mL/s): inflow imposed by peristaltic pump

We tested three values of *θ* parameter: (a) High: 360 s, (b) Medium: 180 s, and (c) Low: 90 s. We have thus three variants of Sc1 corresponding to the three values of *θ* (*s*), and that we note Sc1a, Sc1b, and Sc1c.

Before testing Sc1, we impose a permanent flow condition (*Q_p_* = 3 mL/s) in the channel during 10 min. Then Sc1 is tested for an unsteady flow event having a total duration of 45 min for Sc1a and 35 min for both Sc1b and Sc1c. Sc1 is imposed on the channel input by the peristaltic pump by using the above-mentioned “pump control device”. We replicated Sc1 (with its three variants Sc1a, Sc1b, and Sc1c) three times and then we took the mean of all the replicates (see the results section). 

*Second scenario* (Sc2)

The sine-squared function is given by:

(2) $$Q_{In}\left(t\right)=Q_{p}+A{\left[sin\left(\frac{t}{\tau }\right)\right]}^{2}$$

where

*t* (s): time
*τ* (s): form parameter (taken 115 s)
*A* (mL/s): wave amplitude
*Q_p_* (mL/s): permanent flow regime (taken 2 mL/s)
*Q_In_* (*t*) (mL/s): inflow imposed by peristaltic pump

The form parameter *τ* was chosen in a manner to obtain three successive wave crests during the duration of the Sc2. As to the wave amplitude *A*, we tested three values of this parameter: (a) High: 29 mL/s, (b) Medium: 14.5 mL/s, and (c) Low: 7.25 mL/s. We have thus three variants of Sc2 corresponding to the three values of *A*, and that we note Sc2a, Sc2b, and Sc2c.

Before testing Sc2, we impose a permanent flow condition (*Q_p_* = 2 mL/s) in the channel during 10 min. Then Sc2 is tested for a duration of 30 min for the three variants (Sc2a, Sc2b, and Sc2c). Like Sc1, Sc2 is imposed on the channel input by the peristaltic pump by using the “pump control device”. Each variant of Sc2 was replicated three times and the mean of the replicates is shown in the results section.

## 3. Results and Discussion

**Figure 9:**
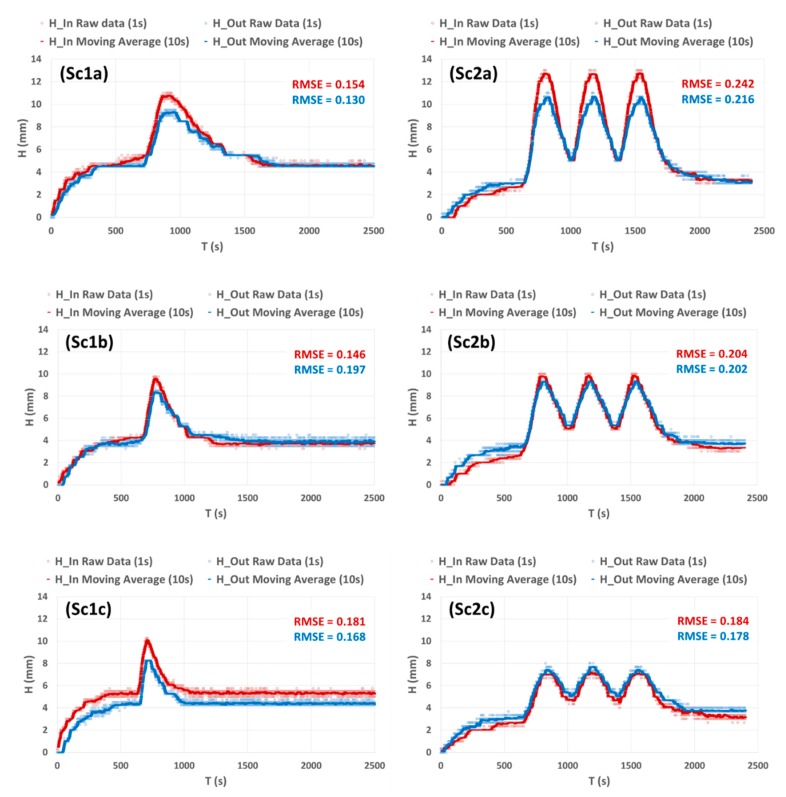
Experimental measurements of WL height at time interval of 1 s for the ensemble of the tested scenarios Sc1a, b, c and Sc2a, b, c. The shown data are the result of three replicates. Light red: WL height at abscissa 0 m (*H_in_*)—raw data at 1 s time interval. Dark red: WL height at abscissa 0 m (*H_in_*)—moving average over 10 s. Light blue: WL height at abscissa 4 m (*H_out_*)—raw data at 1 s time interval. Dark Blue: WL height at abscissa 4 m (*H_out_*)—moving average over 10 s. RMSE (mm) red and blue respectively: root mean squared error between raw data (1 s) and moving average (10 s) for *H_in_* and *H_out_* respectively.

As mentioned in material and method section, the measured outflow curve *Q_Out_* (*t*) and the measured WL curves *H* (*t*) (at different abscissa) are the mean of three replicates. Altough, even with three replicates, the mean curves still present some statistical oscillations because the time interval considered is relatively small (1 s). As shown in the Figure 9, raw data have small oscillations around a clear global behavior. We used the moving average only to replace the ‘step behavior’ of raw data by a smooth and ‘continuous behavior’. In sake of visual clarity, moving average curves will be used in later figures.

The 0.5 cm threshold at the channel output guaranties a minimum WL of 0.5 cm all across the channel. For WL measurements (*H* (*t*) curves), the 0.5 cm threshold is taken as a reference i.e., the origin of height (*H* = 0). 

The Hayami flood function (first scenario—Sc1) was chosen because it is considered to be very close to real river flow behavior during flood events [23]. The θ parameter is the first moment of the Hayami curve. Also, θ is an indicator of the curve dispersion. The more high θ is, the more the flood curve is dispersed in time with its peak occurring for higher time values. Therefore, we obtain sharp unsteady flow events for low θ values and flat unsteady flow events for high θ values. The objective of Sc1 is thus to see to what extent the WL measurement method is able to detect different unsteady flow event dispersions. 

**Figure 10:**
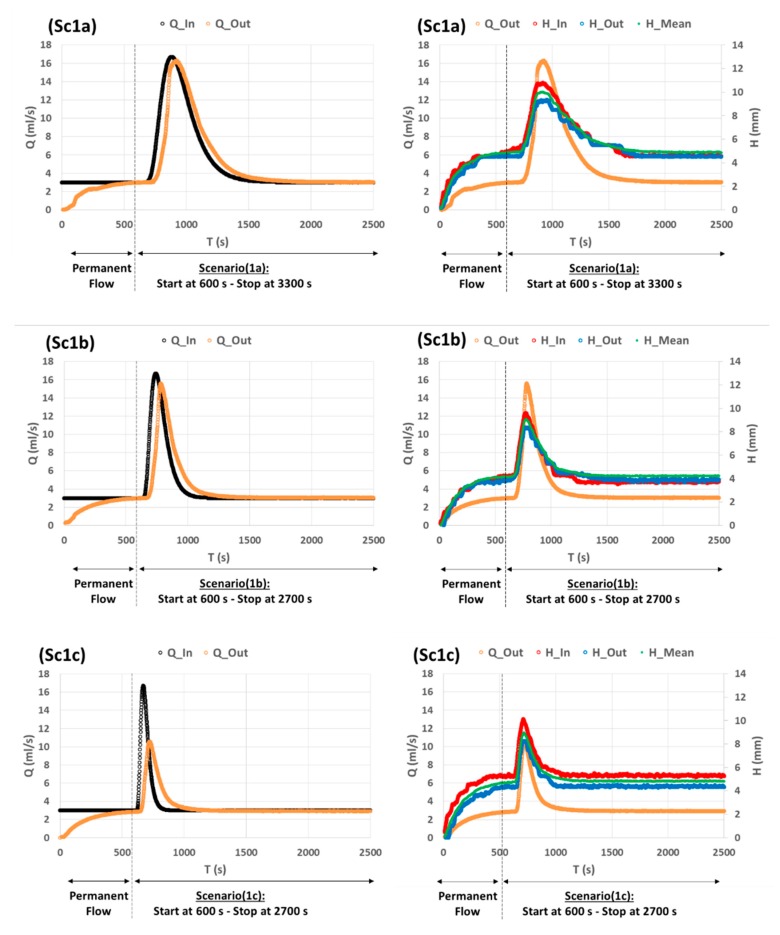
Experimental measurements of Sc1 that represents a Hayami flood function for three values of the *θ* shape parameter: (a) High: 360 s, (b) Medium: 180 s, and (c) Low: 90 s. All the curves are the mean of three replicates with moving average over 10 s. A permanent flow condition is imposed at the channel input during 10 min, then Sc1 is tested during 45 min for Sc1a (from 600 s to 3300 s) and during 35 min for Sc1b,c (from 600 s to 2700 s). Black: Inflow *Q_In_* (*t*) imposed by peristaltic pump. Orange: Outflow *Q_Out_* (*t*) deduced from scales measurements. Red: WL height at abscissa 0 m. Blue: WL height at abscissa 4 m. Green: Mean WL over the ensemble of the channel (estimated from the mean measurements of 8 IR-Sensors).

The results of Sc1 are shown in Figure 10. We see that the WL measurements (*H* (*t*) curves) at the input (abscissa 0 m), at the output (abscissa 4 m) as well as the mean WL (on the ensemble of the channel), follow the inflow variations (*Q_In_* (*t*) curves). In addition, when the *Q_In_* (*t*) curve changes from flat to sharp signal, the *H* (*t*) curves follow the same pattern. This shows that our WL measurement method is sensitive to the dispersion of flow events (with a precision of 1 mm). As to outflow measurements (*Q_Out_* (*t*) curves), they also follow the pattern of *Q_In_* (*t*) curves. We note that for a flat inflow signal, the outflow signal is almost a reproduction of the inflow signal (with a small time delay). Whereas for a sharp inflow signal, the outflow signal is strongly damped, and its time delay is higher (Figure 10, Sc1c). This indicates that, although *H* (*t*) curves and *Q_Out_* (*t*) curves follow the pattern of *Q_In_* (*t*) curves, there is however a difference in the response of *H* (*t*) to *Q_In_* (*t*) and that of *Q_Out_* (*t*) to *Q_In_* (*t*). Therefore, the relation between *H* (*t*) and *Q_Out_* (*t*) is not linear. This is well known in hydrological studies where the stage-discharge rating curves are used to study the relation *Q_Out_* (*H_out_*).

The second scenario (Sc2) is rather a kind of mathematical test not necessarily observed in real world situations—a sine-squared function with three successive wave crests for three different wave amplitudes. The objective of Sc2 is to test the robustness of our WL measurement method by seeing to what extent this method is capable of detecting the successive wave crests passing through the channel.

**Figure 11:**
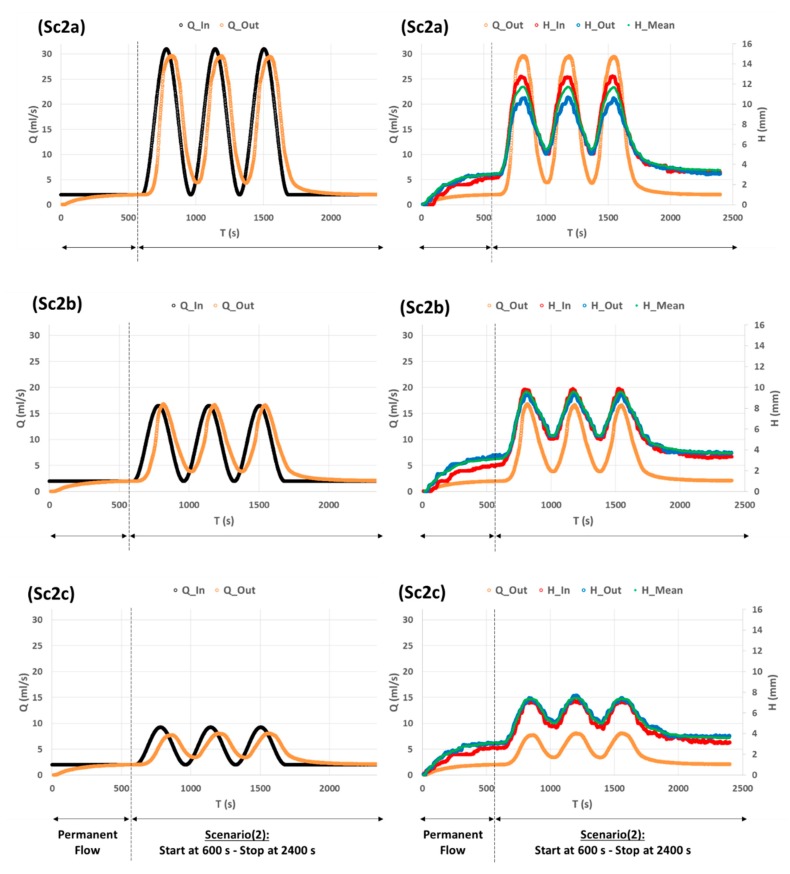
Experimental measurements of Sc2 that represents three wave crests of a sine-squared function with different amplitudes: (a) High: 29 mL/s, (b) Medium: 14.5 mL/s, and (c) Low: 7.25 mL/s. All the curves are the mean of three replicates with moving average over 10 s. A permanent flow condition is imposed at the channel input during 10 min, then Sc2 is tested during 30 min (from 600 s to 2400 s). Black: Inflow *Q_In_* (*t*) imposed by peristaltic pump. Orange: Outflow *Q_Out_* (*t*) deduced from scales measurements. Red: WL height at abscissa 0 m. Blue: WL height at abscissa 4 m. Green: Mean WL over the ensemble of the channel (estimated from the mean measurements of 8 IR-Sensors).

The results of Sc2 are shown in Figure 11. We see that the *H* (*t*) curves follow nicely the pattern of *Q_In_*(*t*) curves and that the successive wave crests passing through the channel are well detected and thus well distinguished, either for high or low wave amplitudes. Our WL measurement method is sensitive to sudden and highly dynamic variations of water surface level. As noticed in the discussion of Sc1 results, we also see in Sc2 results that the response of *H* (*t*) to *Q_In_* (*t*) differs from the response of *Q_Out_* (*t*) to *Q_In_* (*t*). In fact, between Sc2a and Sc2c, the crests of *Q* (*t*) curves drop (roughly) from 29 mL/s to 7 mL/s whereas the crests of *H* (*t*) curves drop from 12 mm to 8 mm. The relative variation of crest drop is 22/29 ≈ 0.75 (respectively 4/12 ≈ 0.33) for *Q* (*t*) curves (respectively *H* (*t*) curves). Therefore, it is interesting to examine how *Q* varies as a function of *H*.

However difficult it may be in real world situation, the measurement of WL in a river is still much easier than the measurement of river flow, which necessitates the exploration of the flux field on the entire river cross-section. Therefore, it is very common to relate river flow (also called discharge) to the WL (also called stage) via a stage-discharge rating curve. Moreover, in classical hydraulics, the discharge is estimated from the stage measurement using theoretical laws such as the Power law or the Maninng-Strickler law.

In order to establish a stage-discharge rating curve on our experimental channel, we took as example the WL at the output (*H_Out_*) and the outflow (*Q_Out_*) from the second (central) crest of the sine-squared function of Sc2a and Sc2c (Figure 11), so that we can compare two situations of *Q_Out_* (*H_Out_*)—a high amplitude flow event in Sc2a, and a low amplitude flow event in Sc2c.

**Figure 12:**
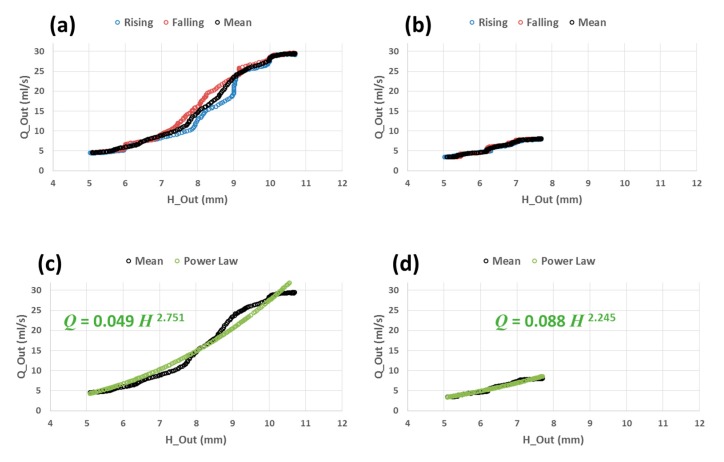
Stage-discharge rating curve relating the outflow *Q_Out_* (mL/s) (estimated by scales measurements) to the WL at the output *H_Out_* (mm) (estimated by IR-Sensor measurement). (**a**) The rating curve is based on the central crest of the sine-squared function of Sc2a. (**b**) The rating curve is based on the central crest of the sine-squared function of Sc2c. Blue: rising WL. Red: falling WL. Black: mean of both rising and falling WL. (**c**) Theoretical adjustment (power law) of mean curve of (**a**). (**d**) Theoretical adjustment (power law) of mean curve of (**b**).

The results of stage-discharge rating curves are shown in Figure 12. We can see that the rating curve is a loop that has two limbs - a rising limb corresponding to rising WL and a falling limb corresponding to falling WL (Figure 12a). It is generally known that rating curves under unsteady flow regime have hysteresis behavior corresponding to rapidly rising or rapidly falling water levels. They are thus called loop rating curves. Our results are consistent with other literature studies [24,25] that noticed loop rating curves in natural rivers under unsteady flow regime.

The rising and falling limbs of the loop rating curve are more distinguishable for higher flow rates (Figure 12a-Sc2a) and they are barely distinguishable for low flow rates (Figure 12b-Sc2c). Since the central crest of the sine-squared function of Sc2 is symmetric, the rising and falling limb have both the same number of points. We can thus calculate their mean (black mean curve in Figure 12) that will serve us to make a theoretical adjustment. It is common to make theoretical adjustment on rating curves. The choice of the theoretical law depends on several parameters: limit condition, channel geometry, slope, and flow rate. 

In our case, we have a threshold at the channel output. We thus made the following power law adjustment:

(3) $$Q_{Out}\left(H_{Out}\right)=\alpha {\left(H_{Out}\right)}^{\beta }$$

where α and β are the adjustment parameters. 

The power law adjustment fits our experimental observation well, and gives an exponent β = 2.751 for Sc2a and β = 2.245 for Sc2c (Figure 12c,d). It is well known in hydraulics that the exponent of the power law adjustment depends on the shape of the threshold. For example, it is 3/2 for rectangular threshold and 5/2 for triangular threshold. The shape of our threshold is a mix of circular and rectangular (Figure 5). For low flow rates, water flows in the circular threshold, whereas for high flow rates, water flows in both circular and rectangular thresholds. The adjustment results give power law exponent between 2.245 and 2.751. This suggests that our threshold behaves globally like a triangular one where the power law exponent is 2.5 (i.e., roughly the mean of 2.245 and 2.751).

**Figure 13:**
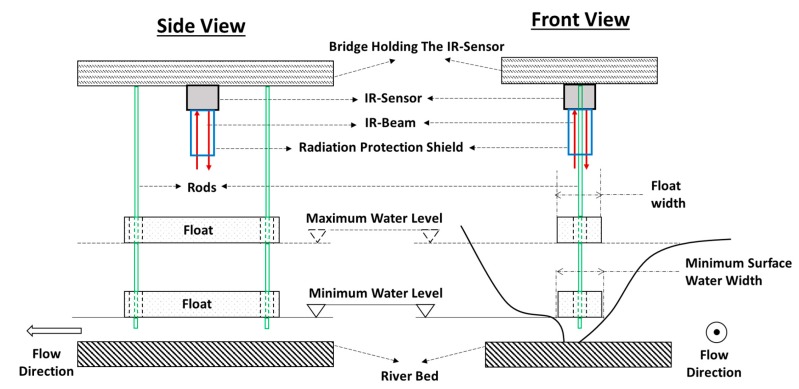
Possible adaptation of the new measurement method to large-scale application (like river) and more complicated cross section geometry (irregular shape). Green: rigid rods passing through the float. Red: infrared beam. Blue: shield that protects the infrared sensor from outdoor radiation. The float width should be equal to the minimum surface water width which corresponds to the minimum water level in the channel.

The new measurement method proposed in this paper could be extended to larger scale applications (like river) and more complicated cross section geometry (irregular shape). In an irregular shape cross section, the use of lateral rods would be inadequate, and the setup needs some modifications. Figure 13 shows one of the ideas that could be applied to keep the float in a standing still position under the IR-Sensor in a large-scale application and in an irregular cross section channel.

The rods should be rigid and fixed to the bridge. The floats should be perforated at their extremities (cylindrical perforation) so that the rods penetrate the perforations with no friction. Therefore, when the water level varies, the float can make vertical displacements along the rods by staying at a standing still position under the IR-Sensor. 

To adapt this setup to a channel cross section with irregular shape, the width of the float should be small enough to cover the range of minimum to maximum water level without touching the boundaries of the cross section. This condition would be guaranteed if the float width is set to the minimum surface water width corresponding to the minimum water level (or threshold) of the channel. 

To adapt this setup to outdoor conditions, the IR-Sensor cell should be protected from outdoor radiation, whether it comes directly from the sun or indirectly by reflection. This could be done by installing a shield (cylinder, for example) which is long enough to guaranty the transmission of only vertical infrared beams coming from the IR-Sensor emitter and going back to the IR-Sensor receiver. Any outdoor radiation beam would have a slight angle with the vertical, and it will not be transmitted to the IR-Sensor due to the shield. Moreover, as the infrared sensor may be sensitive to temperature variations, the effect of air temperature should be taken into account. Temperature measurements should be coupled with IR-Sensor measurements in the same electronic device, and the WL measurements given by the IR-Sensor should be rectified/corrected according to a pre-established correlation that relates the variations of IR-Sensor distance measurement to the variations of air temperature.

Finally, the bridge holding the IR-Sensor could be an already existing bridge, but it could also be an easy to use metallic structure that can be installed and removed on any location at the river border. The mobility of the bridge structure would make the measurement method (IR-sensor + float) a powerful tool for the assessment of accurate WL measurements on different space locations.

## 4. Conclusions

In this study, we were interested in measuring water level variation during unsteady flow events in an experimental channel (4 m in length, 3 cm in width). The channel is designed to study flow scenarios where the surface water level (WL) can vary between 0 and 2 cm. Therefore, we needed a WL measurement method that has a detection precision of 1 mm for WL variations in a range of 2 cm. After several preliminary tests, we realized that the existing WL detection techniques could not meet our measurement requirements. Therefore, we proposed a new measurement method that combines two approaches: an “old” water contact technique (float) with a “new” remote non-contact technique (infrared sensor). We used an extruded polystyrene (XPS foam) that needed some adequate treatment before using it as float in experimental measurements. The combination of IR-sensors with treated float foam lead to a sensitive measurement method (with a precision of 1 mm) that is able to detect flat and sharp flow signals (Hayami function) as well as highly dynamic variations of water surface level (sine-squared function). Based on the experimental measurements of WL and the outflow at the channel output (for various types of unsteady flow events), we deduced a loop rating curve which has rising and falling limbs. The loop rating curve was suitably adjusted by a power law function. This experimental channel will serve as a platform for testing experimental scenarios of unsteady flow regime to be used in hydraulic/hydrological modelling. Finally, the new proposed measurement method could be extended to larger scale applications like rivers and more complicated cross section geometry of irregular shape.

## References

[1] Jung S., Kang J., Hong I., Yeo H. (2012). Case Study: Hydraulic Model Experiment to Analyze the Hydraulic Features for Installing Floating Islands. Engineering.

[2] Ettema R., Muste M. (2004). Scale Effects in Flume Experiments on Flow around a Spur Dike in Flatbed Channel. J. Hydraul. Eng..

[3] Defina A., Peruzzo P. (2010). Floating particle trapping and diffusion in vegetated open channel flow. Water Resour. Res..

[4] Vinatier F., Bailly J.S., Belaud G. (2017). From 3D grassy vegetation point cloud to hydraulic resistance: Application to close-range estimation of Manning coefficients for intermittent open channels. Ecohydrology.

[5] Zheng G., Zong H., Zhuan X., Wang L. (2010). High-accuracy surface-perceiving water level gauge with self-calibration for hydrography. IEEE Sens. J..

[6] Duraibabu D.B., Leen G., Toal D., Newe T., Lewis E., Dooly G. (2017). Underwater depth and temperature sensing based on fiber optic technology for marine and fresh water applications. Sensors.

[7] Teng C., Liu H., Deng H., Deng S., Yang H., Xu R., Chen M., Yuan L., Zheng J. (2018). Liquid level sensor based on a V-groove structure. Sensors.

[8] Yoo W.J., Sim H.I., Shin S.H., Jang K.W., Cho S., Moon J.H., Lee B. (2014). A fiber-optic sensor using an aqueous solution of sodium chloride to measure temperature and water level simultaneously. Sensors.

[9] Zhang Y., Zhang Y., Hou Y., Zhang L., Hu Y., Gao X., Zhang H., Liu W. (2018). An optical fiber liquid level sensor based on side coupling induction technology. J. Sens..

[10] Da Silva J.S., Calmant S., Seyler F., Filho O.C.R., Cochonneau G., Mansur W.J. (2010). Water levels in the Amazon basin derived from the ERS 2 and ENVISAT radar altimetry missions. Remote Sens. Environ..

[11] Fréville P., Montoux N., Baray J.L., Chauvigné A., Réveret F., Hervo M., Dionisi D., Payen G., Sellegri K. (2015). LIDAR developments at Clermont-Ferrand—France for atmospheric observation. Sensors.

[12] Dimitrievski M., Veelaert P., Philips W. (2019). Behavioral pedestrian tracking using a camera and LIDAR sensors on a moving vehicle. Sensors.

[13] Vaquero V., Repiso E., Sanfeliu A. (2019). Robust and real-time detection and tracking of moving objects with minimum 2D LIDAR information to advance autonomous cargo handling in ports. Sensors.

[14] Shen K., Lu H., Baig S.A., Jiang G., McManus J.W., Wang M.R. (2017). Laser-based water depth measurement system deployed via unmanned aerial vehicle. J. Appl. Remote Sens..

[15] Kruger A., Krajewski W.F., Niemeier J.J., Ceynar D.L., Goska R. (2016). Bridge-Mounted river Stage Sensors (BMRSS). IEEE Access.

[16] Moreno C., Aquino R., Ibarreche J., Pérez I., Castellanos E., Álvarez E., Rentería R., Anguiano L., Edwards A., Lepper P. (2019). RiverCore: IoT device for river water level monitoring over cellular communications. Sensors.

[17] Mousa M., Zhang X., Claudel C. (2016). Flash flood detection in urban cities using ultrasonic and infrared sensors. IEEE Sens. J..

[18] Arattano M., Marchi L. (2008). Systems and sensors for debris-flow monitoring and warning. Sensors.

[19] Lo S.W., Wu J.H., Lin F.P., Hsu C.H. (2015). Cyber surveillance for flood disasters. Sensors.

[20] Turner D., Lucieer A., Watson C. (2012). An automated technique for generating georectified mosaics from ultra-high resolution Unmanned Aerial Vehicle (UAV) imagery, based on structure from motion (SfM) point clouds. Remote Sens..

[21] Casella E., Collin A., Harris D., Ferse S., Bejarano S., Parravicini V., Hench J.L., Rovere A. (2017). Mapping coral reefs using consumer-grade drones and structure from motion photogrammetry techniques. Coral Reefs.

[22] Le Boursicaud R., Pénard L., Hauet A., Thollet F., Le Coz J. (2016). Gauging extreme floods on YouTube: Application of LSPIV to home movies for the post-event determination of stream discharges. Hydrol. Process..

[23] Hayami S. (1951). On the Propagation of Flood Waves.

[24] Braca G. (2008). Stage–Discharge Relationships in Open Channels: Practices and Problems.

[25] Kuhnle R.A., Bowie A.J. (1992). Loop Rating Curves from Goodwin Creek.

